# Supplementary material for: Splice-Junction-Based Mapping of Alternative Isoforms in the Human Proteome
Source: Cell Rep. Author manuscript; Available in PMC 2020 Jan 15. (PMC6961840; doi:10.1016/j.celrep.2019.11.026)
Supplement: 3 [file NIHMS1546469-supplement-3.zip › DF2/PXD006675/PulmonaryArtery-153-P52272-INGGGGGSVPGIER.pdf]

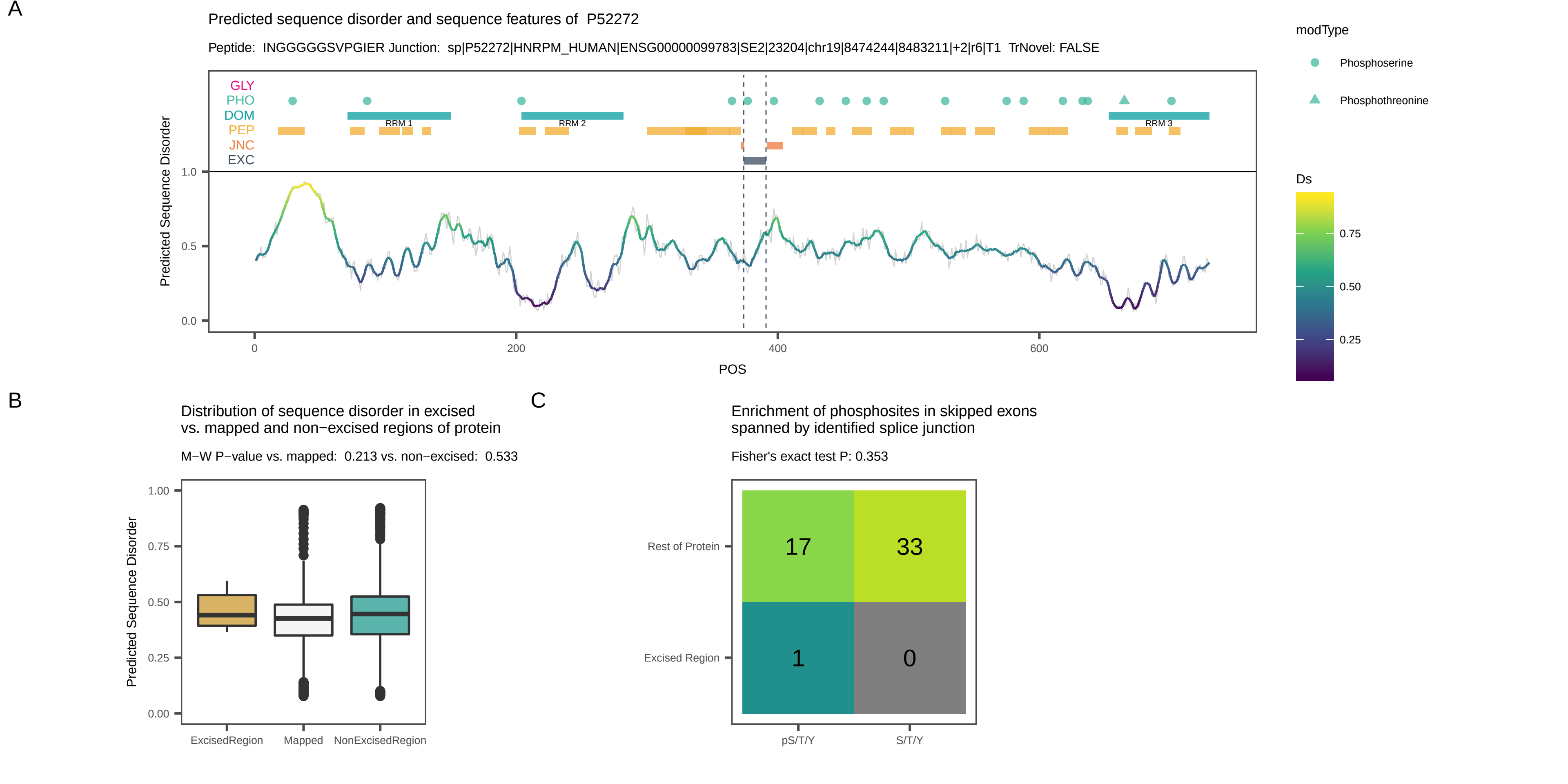
A
Predicted sequence disorder and sequence features of P52272
Peptide: INGGGGGGSVPGIER Junction: sp|P52272|HNRPM_HUMAN|ENSG00000099783|SE2|23204|chr19|8474244|8483211|+2|r6|T1 TrNovel: FALSE
GLY
PHO
DOM
PEP
JNC
EXC
RRM 1
RRM 2
RRM 3
Predicted Sequence Disorder
POS
modType
Phosphoserine
Phosphothreonine
Ds
B
Distribution of sequence disorder in excised vs. mapped and non−excised regions of protein
M−W P−value vs. mapped: 0.213 vs. non−excised: 0.533
Predicted Sequence Disorder
ExcisedRegion
Mapped
NonExcisedRegion
C
Enrichment of phosphosites in skipped exons spanned by identified splice junction
Fisher's exact test P: 0.353
Rest of Protein
Excised Region
17
33
1
0
pS/T/Y
S/T/Y
